# Weight-bearing or non-weight-bearing after surgical treatment of ankle fractures: a multicenter randomized controlled trial

**DOI:** 10.1007/s00068-018-1016-6

**Published:** 2018-09-24

**Authors:** Diederik Pieter Johan Smeeing, Roderick Marijn Houwert, Jan Paul Briet, Rolf Hendrik Herman Groenwold, Koen Willem Wouter Lansink, Luke Petrus Hendrikus Leenen, Peer van der Zwaal, Jochem Maarten Hoogendoorn, Mark van Heijl, Egbert Jan Verleisdonk, Michiel Joseph Marie Segers, Falco Hietbrink

**Affiliations:** 1 Department of Surgery, St. Antonius Hospital Nieuwegein, PO Box 2500, 3430 EM Nieuwegein, The Netherlands; 2 Utrecht Traumacenter, Utrecht, The Netherlands; 3 Department of Surgery, University Medical Center Utrecht, Utrecht, The Netherlands; 4 Department of Surgery, Diakonessenhuis Utrecht, Utrecht, The Netherlands; 5 Department of Epidemiology, Julius Center for Health Sciences and Primary Care, University Medical Center Utrecht, Utrecht, The Netherlands; 6 Department of Surgery, Elisabeth-TweeSteden Hospital, Tilburg, The Netherlands; 7 Department of Orthopaedics, Haaglanden Medisch Centrum, The Hague, The Netherlands; 8 Department of Surgery, Haaglanden Medisch Centrum, The Hague, The Netherlands

**Keywords:** Ankle fracture, Postoperative care, Weight-bearing, Mobilization, Randomized controlled trial

## Abstract

**Purpose:**

The goal of this study was to assess if unprotected weight-bearing as tolerated is superior to protected weight-bearing and unprotected non-weight-bearing in terms of functional outcome and complications after surgical fixation of Lauge-Hansen supination external rotation stage 2–4 ankle fractures.

**Methods:**

A multicentered randomized controlled trial was conducted in patients ranging from 18 to 65 years of age without severe comorbidities. Patients were randomized to unprotected non-weight-bearing, protected weight-bearing, and unprotected weight-bearing as tolerated. The primary endpoint of the study was the Olerud Molander Ankle Score (OMAS) 12 weeks after randomization. The secondary endpoints were health-related quality of life using the SF-36v2, time to return to work, time to return to sports, and the number of complications.

**Results:**

The trial was terminated early as advised by the Data and Safety Monitoring Board after interim analysis. A total of 115 patients were randomized. The O’Brien–Fleming threshold for statistical significance for this interim analysis was 0.008 at 12 weeks. The OMAS was higher in the unprotected weight-bearing group after 6 weeks c(61.2 ± 19.0) compared to the protected weight-bearing (51.8 ± 20.4) and unprotected non-weight-bearing groups (45.8 ± 22.4) (*p* = 0.011). All other follow-up time points did not show significant differences between the groups. Unprotected weight-bearing showed a significant earlier return to work (*p* = 0.028) and earlier return to sports (*p* = 0.005). There were no differences in the quality of life scores or number of complications.

**Conclusions:**

Unprotected weight-bearing and mobilization as tolerated as postoperative care regimen improved short-term functional outcomes and led to earlier return to work and sports, yet did not result in an increase of complications.

**Electronic supplementary material:**

The online version of this article (10.1007/s00068-018-1016-6) contains supplementary material, which is available to authorized users.

## Introduction

Ankle fractures are the most common type of lower extremity injury and the incidence is rising [6, 8]. More than half of ankle fractures are supination external rotation fractures (classified according to the Lauge-Hansen classification), caused by minor trauma [7, 16]. For a good functional recovery and prevention of osteoarthritis, fractures with incongruency of the tibiotalar joint are treated operatively [19]. Subsequently, a wide variety of postoperative care regimens are used, ranging from immobilization in a cast without weight-bearing to immediate weight-bearing without a cast [11, 31, 35]. Various studies have shown good functional outcomes 1 year after surgery for the different postoperative protocols [9, 26, 35]. However, immobilization in a cast can result in stiffness of the ankle and non-weight-bearing can result in delayed functional recovery [9, 22, 35]. Protective devices such as a brace have shown high rates of postoperative wound complications, with a potentially decreased functional outcome [17, 25].

Even more, postoperative treatment regimens might influence the efficacy of functional recovery, as differences in the time to return to work between the various treatment regimens are suggested [28]. Therefore, these regimes have a direct impact on societal costs [28]. However, due to heterogeneity of studies, it remains unclear which postoperative treatment regimen is preferable and whether specific types of patients or fractures might benefit from a more progressive regimen. Recently, unprotected weight-bearing was described in a small cohort study, which suggested a significant earlier return to work without a higher rate of complications compared to protected non-weight-bearing [11]. Biomechanical analysis has shown no implant failure or loss of reduction after early weight-bearing of surgically treated ankle fractures [32]. However, the functional outcome and the safety of unprotected early weight-bearing and mobilization remain unclear, as the combination of both early weight-bearing and mobilization has not been studied in a randomized trial [9, 18].

Prior studies included a wide range of ankle fractures, including those with syndesmotic injuries [10, 17, 33]. This poses a challenge, as different types of ankle fractures may require a different operative and postoperative approach. In addition, most studies did not employ strict patient selection with respect to comorbidities that may affect rehabilitation [17, 33].

The objective of this multicenter randomized controlled trial was to provide evidence for the optimal postoperative treatment regimen after surgical fixation of Lauge-Hansen supination external rotation stage 2–4 ankle fractures [5]. The goal of this study was to assess if unprotected weight-bearing as tolerated is superior to protected weight-bearing and unprotected non-weight-bearing in terms of functional outcome and complications.

## Materials and methods

### Trial design and population

The WOW study, registered in the Dutch Trial Register (NTR3727), was performed in accordance with the Declaration of Helsinki and approved by the local Institutional Review Board (CCMO) under protocol number WOW-01/NL40835.100.12 [36]. The rationale and methods are described in more detail in the published study protocol [5]. No changes to the protocol occurred after trial commencement.

**Fig. 1:**
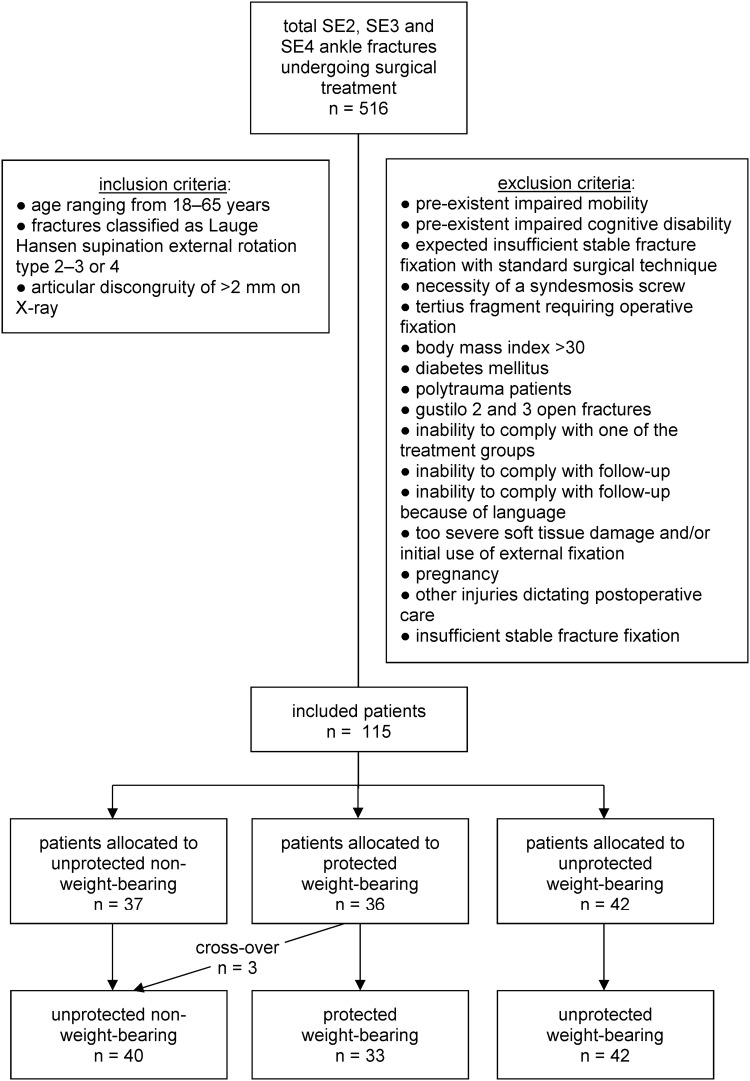
Flowchart of patient selection in the WOW! Study. Flowchart indicating patient selection. Three patients crossed over due to cast irritation. *SE* Supination external rotation

Patients with a supination external rotation type2–4 ankle fracture were included between January 2013 and October 2016 [16, 23]. In- and exclusion criteria are shown in Fig. 1. Patients were recruited and enrolled at the emergency departments or outpatient clinics of the participating hospitals in an urban environment. Indication for surgery was stated at the discretion of the treating surgeon. The study was conducted in four teaching hospitals in the Netherlands: Diakonessenhuis (level2 traumacenter), St. Antonius Hospital (level2 traumacenter), Haaglanden Medisch Centrum (level1 traumacenter) and the Elisabeth-TweeSteden hospital (level1 traumacenter).

In order for a participant to be included in the trial, informed consent had to be obtained and the fracture had to be classified by at least three out of six orthopedic trauma surgeons as a supination external rotation injury [5]. The expert panel consisted of six experienced orthopedic trauma surgeons. The expert panel was notified about a potential participant by an email that included an anonymous (non-stress) X-ray for classification on a web-based database. A patient was eligible for inclusion when the majority of the panel agreed on the Lauge-Hansen fracture type and all other inclusion criteria were met. When votes were split equally, the chairman’s (LL) vote was decisive. The operative technique and implants used were dictated by surgeon preference and the surgeon had a final decision in participant enrollment after testing the syndesmosis intra-operatively.

### Randomization and postoperative treatment

Patients were randomized using a computerized random number generator with an allocation ratio of 1:1:1 in blocks of 21 patients. The three postoperative care regimens were: (1) unprotected non-weight-bearing—mobilization with crutches, active ankle exercises without cast or brace, (2) protected weight-bearing—weight-bearing as tolerated with a below knee cast for 6 weeks, or (3) unprotected weight-bearing—functional weight-bearing as tolerated. Unprotected non-weight-bearing included a pressure dressing in the first 24 h postoperatively. After 6 weeks, weight-bearing was allowed. Protected weight-bearing included a 10-day back slab plaster splint. After 10 days, the patient received a weight-bearing cast for the remaining first 6 weeks. Unprotected weight-bearing included a pressure dressing in the first 24 h postoperatively. After the first 24 h, weight-bearing was allowed as tolerated by the patient. All patients consulted a physical therapist postoperatively or after cast removal to learn exercises and received advice on how to start mobilizing according to their specific postoperative care regimen.

### Endpoints and follow-up

The primary endpoint of the study was the functional outcome using the Olerud Molander Ankle Score (OMAS) at 12 weeks after randomization [24]. The OMAS is a patient-reported outcome measure for evaluating symptoms after ankle injuries. The OMAS ranges from 0 to 100. A higher score indicates a better functional outcome [24]. The secondary endpoints were health-related quality of life using the SF-36v2, time to return to work in weeks, time to the return to sports in weeks, and the number of complications [1]. A higher score of the SF-36v2 indicates less disability (range from 0 to 100). Follow-up was performed at 6 weeks, 12 weeks, 6 months and 1 year after randomization. A coordinating researcher was in charge of all follow-up visits to prevent inconsistency of data.

### Sample size calculations

A sample size calculation was performed for a superiority trial of treatment with unprotected weight-bearing versus protected weight-bearing versus unprotected non-weight-bearing, based on a previous report covering protected weight-bearing or ankle exercises [28]. To detect a clinically significant 7-point difference (SD10) on the OMAS at 12 weeks, with two-sided *α* = 0.05 and power of 0.90, a sample size of 60 subjects per treatment arm was required (i.e., 180 subjects in total). We anticipated a maximum loss to follow-up of 20%, leading to a sample size of 75 patients per group and a total of 225 patients needed for this study [5].

### Statistical analysis and interim analysis

Patients were analyzed in accordance with the intention-to-treat principal. Continuous data were presented as means with standard deviations; dichotomous data as frequencies with percentages. Continuous outcome data (OMAS, time to return to work, time to return to sports, and the SF-36) were compared by means of ANOVA; dichotomous outcome data (complications) were compared by means of Pearson’s Chi square test. At 3 months and 1 year, follow-up information was available for 88% and 86% of the subjects, respectively, for all outcomes. We performed an available case analysis, and no corrections for loss to follow-up were applied. No subgroup or other additional analyses were performed. All statistical analysis were performed using Statistical Package for the Social Sciences (IBMCorp. Released 2016. IBMSPSS Statistics for Windows, Version 24.0. Armonk, NY:IBMCorp.).

An interim analysis was planned after every serious adverse event and after half of the target enrollment was reached. A data and safety monitoring board (DSMB), consisting of two independent physicians and one epidemiologist, was established to advise the steering committee. The study would be terminated early if (1) 10% hardware failure occurred in any of the treatment groups, (2) wound infection percentages exceeded 20% in any of the treatment groups, or (3) statistically significant, sufficiently powered, and clinically relevant results were reached at interim analysis [5]. The post hoc O’Brien–Fleming (OBF) alpha spending function was used, to determine the statistical significance level based on the proportion of subjects included in the interim analysis to the required sample size in the absence of loss to follow-up.

### Source of funding

No funding was received for this study.

## Results

### Patient selection and baseline characteristics

A total of 115 consecutive patients were included at interim analysis. The OBF threshold for statistical significance of the primary outcome for this interim analysis was 0.008. Figure 1 shows a flowchart of the selection of patients, including the number of excluded patients. Appendix 1 shows the number of patients who were not considered eligible for participation with the reasons for exclusion. The baseline characteristics of the patients who were included in the trial are shown in Table 1. The study also included patients with (SE-4) fracture dislocations. The mean age of the included patients was 39.0 (± 14.4) years, ranging from 18 to 65 years, of which 61 (53.0%) were men. All surgeries were performed or supervised by consultant orthopedic trauma surgeons. Implants used are shown in Table 1. A total of three patients switched from the protected weight-bearing group to the unprotected non-weight-bearing group because of cast irritation. Follow-up rates of the participating hospitals are described in Appendix 2.

**Table 1:** Baseline characteristics of included patients with ankle fracture

	Unprotected non-weight-bearing (*n* = 37)	Protected weight-bearing (*n* = 36)	Unprotected weight-bearing (*n* = 42)
Mean age in years (± SD)	37.8 (± 13.7)	41.5 (± 14.2)	37.8 (± 15.1)
Age range in years	18–65	18–62	18–65
Number of males (%)	23 (62.2%)	16 (44.4%)	22 (52.4%)
Symptomatic side right	21 (56.8%)	23 (63.9%)	18 (42.9%)
Smoking	7 (28.0%) (12 missing)	5 (22.7%) (14 missing)	9 (36.0%) (17 missing)
Cause of fracture			
Fall < 3 m	0 (%)	2 (5.6%)	4 (9.5%)
Fall from scooter	0 (%)	0	2 (4.8%)
Fall from bicycle	8 (21.6%)	8 (22.2%)	8 (19.0%)
Sport	9 (24.3%)	3 (8.3%)	13 (31.0%)
Winter sports	1 (2.7%)	2 (5.6%)	1 (2.4%)
Simple inversion	17 (45.9%)	20 (55.6%)	12 (28.6%)
Other	2 (5.4%)	1 (2.8%)	2 (4.8%)
Lauge-Hansen classification			
Supination external rotation type 2	14 (37.8%)	9 (25.0%)	14 (33.3%)
Supination external rotation type 3	2 (5.4%)	4 (11.1%)	0
Supination external rotation type 4	21 (56.8%)	23 (63.9%)	28 (66.7%)
Type of fixation lateral malleolus			
2× screw fixation	3 (8.1%)	4 (11.1%)	7 (16.7%)
Screw fixation and neutralization plate	19 (51.4%)	22 (61.1%)	23 (54.8%)
Neutralization plate	7 (18.9%)	7 (19.4%)	6 (14.3%)
Dorsolateral buttress plate	8 (21.6%)	3 (8.3%)	6 (14.3%)
Type of fixation medial malleolus			
1× screw fixation	1 (2.7%)	2 (5.6%)	1 (2.4%)
1× screw fixation and Kirschner wire	2 (5.4%)	0	4 (9.5%)
2× screw fixation	2 (5.4%)	1 (2.8%)	1 (2.4%)
Zuggertung	2 (5.4%)	1 (2.8%)	6 (14.3%)
Other	0	1 (2.8%)	0
Not applicable/no fractured medial malleolus/no fixation required	30 (81.1%)	31 (86.1%)	30 (71.4%)

No statistically significant differences were found in baseline characteristics between the three treatment groups after randomization

*SD* Standard deviation

### Primary outcome: functional outcome scores

**Fig. 2:**
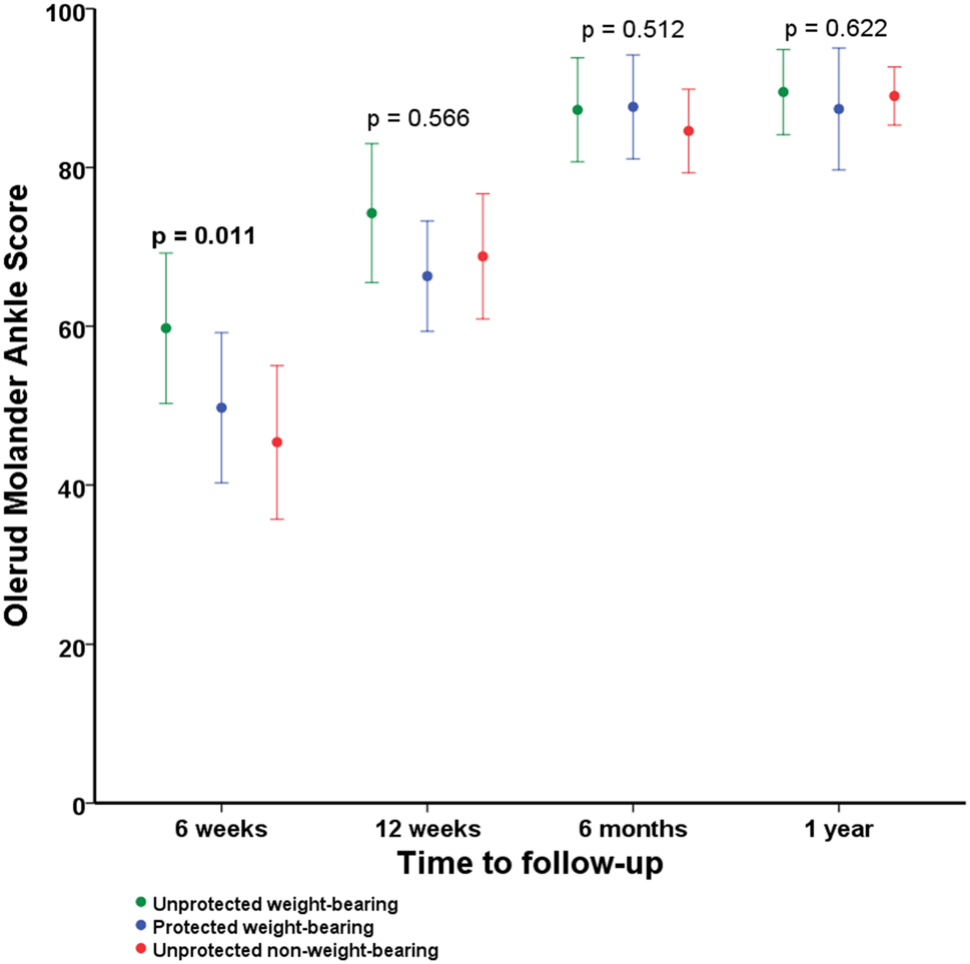
Functional outcome using the Olerud Molander Ankle Score based on the postoperative treatment of ankle fractures. This figure shows the functional outcome using the Olerud Molander Ankle Score based on the postoperative treatment of ankle fractures. The Olerud Molander Ankle Score ranges from 0 to 100. A higher score indicates a better functional outcome. Mean Olerud Molander Ankle Scores are shown with corresponding 95% confidence intervals of the means on the different time points

The OMAS was higher in the unprotected weight-bearing group after 6 weeks (61.2 ± 19.0) compared to the protected weight-bearing (51.8 ± 20.4) and unprotected non-weight-bearing groups (45.8 ± 22.4) (*p* = 0.011) (Table 2; Fig. 2). All other follow-up time points did not show significant differences in terms of OMAS between the groups. Appendix 3 provides details about the pairwise comparisons of the functional outcomes between the three groups.

**Table 2:** Results and differences in Olerud Molander Ankle Score, time to return to work and sports

	Unprotected non-weight-bearing (*n* = 37)	Protected weight-bearing (*n* = 36)	Unprotected weight-bearing (*n* = 42)	*p* value
Olerud Molander score 6 weeks	45.8 (± 22.4)	51.8 (± 20.4)	61.2 (± 19.0)	0.011*
Olerud Molander score 12 weeks	67.9 (± 19.8)	68.6 (± 14.6)	72.2 (± 19.4)	0.566*
Olerud Molander score 6 months	80.9 (± 18.0)	86.0 (± 13.9)	85.5 (± 19.2)	0.496*
Olerud Molander score 1 year	88.7 (± 11.4)	89.1 (± 15.0)	86.8 (± 16.0)	0.773*
Time to return to work in weeks˟	7.0 (± 5.3) (*n* = 32)	5.7 (± 4.9) (*n* = 34)	4.1 (± 3.3) (*n* = 39)	0.028*
Time to return to sports in weeks˟	14.1 (± 5.7) (*n* = 26)	12.7 (± 8.4) (*n* = 25)	8.9 (± 4.7) (*n* = 35)	0.005*
Complications	Total: 5 (13.5%) − 3× low grade infection° − 1× dystrophy − 1× deep venous thrombosis	Total: 1 (2.7%) − 1× low grade infection°	Total: 3 (7.1%) − 3× low grade infection°	0.228^†^

This table shows the functional outcome using the Olerud Molander Ankle Score, the time to return to work and sports, and the number of complications based on the postoperative treatment of ankle fractures. The Olerud Molander Ankle Score ranges from 0 to 100. A higher score indicates a better functional outcome. Improved function was seen at 6 weeks in the mobilization as tolerated study arm. On all other time points no significant differences were seen. In addition, patients in the unprotected weight-bearing group demonstrated a reduced time to return to work and sports

Mean scores (± standard deviation) are shown

*ANOVA

˟Analysis was performed on the number of patients that reported they had work, respectively, performed sports

°A low-grade infection was defined as a clinical suspicion of a wound infection based on redness and pus and/or fever in combination with the necessity of antibiotic treatment

^†^Chi square

### Secondary outcomes: time to return to work, time to return to sports, quality of life and complications

Unprotected weight-bearing showed a significantly earlier return to work with a mean of 4.1 weeks compared to 5.7 and 7.0 weeks (*p* = 0.028). The patients in the unprotected weight-bearing group also showed an earlier return to sports with a mean of 8.9 weeks compared to 12.7 and 14.1 weeks (*p* = 0.005). Radiographic consolidation was reached in all fractures. There were no significant differences in quality of life scores between the different groups after both 12 weeks and 1 year (Table 3). There were no significant differences in the rate of complications between groups (*p* = 0.639). The number and type of complications are shown in Table 2 for each group.

**Table 3:** Results and differences in quality of life using the SF-36

	Unprotected non-weight-bearing (*n* = 37)	Protected weight-bearing (*n* = 36)	Unprotected weight-bearing (*n* = 42)	*p* value*
SF-36 score at 12 weeks				
Physical functioning	74.9 (± 22.8)	75.5 (± 18.3)	76.8 (± 21.9)	0.922
Role-physical functioning	41.7 (± 41.8)	31.9 (± 37.7)	40.8 (± 40.4)	0.575
Bodily pain	76.9 (± 18.7)	76.1 (± 15.0)	73.2 (± 22.2)	0.696
General health perception	80.9 (± 17.8)	78.1 (± 18.5)	77.6 (± 13.2)	0.676
Energy/fatigue (vitality)	73.9 (± 20.5)	72.2 (± 14.1)	69.9 (± 18.0)	0.629
Social functioning	83.3 (± 18.7)	80.6 (± 21.0)	79.0 (± 23.4)	0.686
Role-emotional functioning	15.2 (± 31.3)	11.5 (± 27.1)	14.9 (± 31.7)	0.869
Mental health	83.3 (± 14.1)	85.5 (± 11.5)	81.1 (± 15.8)	0.439
SF-36 score at 1 year				
Physical functioning	93.1 (± 10.0)	90.5 (± 16.3)	88.8 (± 17.1)	0.459
Role-physical functioning	5.7 (± 17.2)	16.7 (± 33.4)	19.1 (± 37.0)	0.150
Bodily pain	92.1 (± 16.4)	86.4 (± 20.4)	84.7 (± 21.7)	0.249
General health perception	81.4 (± 13.7)	80.5 (± 17.3)	79.2 (± 17.9)	0.846
Energy/fatigue (vitality)	72.1 (± 16.1)	73.8 (± 16.2)	75.5 (± 17.1)	0.682
Social functioning	90.4 (± 18.5)	93.9 (± 12.1)	91.5 (± 19.1)	0.675
Role-emotional functioning	6.7 (± 22.6)	12.1 (± 31.0)	17.5 (± 37.0)	0.328
Mental health	80.7 (± 14.2)	81.9 (± 13.6)	81.6 (± 16.8)	0.939

This table shows the health-related quality of life using the SF-36v2 based on the postoperative treatment of ankle fractures. A higher score of the SF-36v2 indicates less disability (range from 0 to 100). No significant differences were seen

Mean scores (± standard deviations) are shown

*ANOVA

## Discussion

The main message of this study is “get up and walk”. Orthopedic trauma surgeons tend to be careful with weight-bearing after surgical treatment of extremity injuries to avoid loss of reduction and implant failure. As these injuries occur often in the working population, this attitude has tremendous socio-economic implications. The patient and society might benefit from a more liberal approach towards early mobilization after ankle fracture surgery. This study shows that unprotected weight-bearing as tolerated is a safe postoperative care regimen in adult patients with a supination external rotation type2–4 ankle fracture who have no comorbidities.

The study methodology with a prospective expert panel is new in orthopedic trauma surgery. The expert panel confirms the fracture type prior to study inclusion. In contemporary literature, mostly an anatomic determination (“ankle”) is used, instead of a specific fracture type [9–11]. The strict selection of fracture type as used in the present study (SE) is essential, as different types of ankle fractures require a different operative and postoperative approach. Consequently, results of this study do not generalize to patients with other fracture types. This unique method of selecting fractures by a classification-guided expert panel helps to identify the right patient population in which a postoperative treatment regimen may be effective [3, 34]. As such, it prevents the inclusion of an overly heterogeneous patient population in whom treatment effects are equally heterogeneous. By ensuring a specific fracture type, a more solid advice regarding effectiveness of treatment can be obtained [29, 30]. We believe this approach of targeted treatment in carefully selected patients should be the new standard for studies in orthopedic trauma surgery.

Over time, the postoperative care regimens of injuries and particularly fractures have evolved towards favoring early weight-bearing and/or mobilization to optimize fast and functional recovery. In addition, several studies on different lower extremity fractures did not demonstrate negative effects of early weight-bearing [12, 13, 15]. However, factors such as comorbidities and injury pattern, influence the surgeon’s choice of postoperative care regimen in an attempt to avoid complications [31]. With respect to the postoperative treatment of ankle fractures, multiple studies investigated either early weight-bearing or early mobilization (active ankle exercises), but not a combination [9, 10, 26, 35]. Previously, early protected weight-bearing showed good functional outcomes without disadvantages [9, 26]. In addition, early mobilization showed an earlier return to work [10, 35]. One retrospective cohort study examined unprotected weight-bearing and mobilization as tolerated, which revealed a significantly earlier return to work without a higher rate of complications [11]. When compared to other studies, our 6-week OMAS results of the early protected weight-bearing group are similar to the comparable group in the study by Dehghan et al. However, the results of our unprotected weight-bearing group was 19 points higher on the 6-week OMAS compared to the protected early weight-bearing group in the study by Dehghan et al. [9]. Hence, our study not only supports the results of Gul et al. given an earlier return to work, but also demonstrates an earlier return to sports and improved functional outcome 6 weeks postoperatively for mobilization as tolerated. In addition, several remarkable observations were logged in the mobilization as tolerated group (Appendix 4).

The study protocol incorporated strict criteria for early termination of the trial due to complications [5]. An earlier retrospective cohort study in our region showed a 10% wound complication rate and a 4% implant failure rate [27]. The results of this study showed that wound-related complications, our primary outcome, had a multifactorial origin but were not associated with the different postoperative treatment protocols. This is in contrast to Keene et al., in which early mobilization resulted in a higher rate of infection compared to immobilization and a study of Lehtonen et al. that even suggested an immobilization period of 2 weeks to reduce infectious complications [14, 17, 27]. To prevent confounders to influence the rate of postoperative complications, the trial enforced strict in- and exclusion criteria concerning both the patient and trauma mechanism [5]. This may have contributed to the lower rate of complications as compared to the historical cohort study and the above-mentioned studies [17, 27].

Early termination is not unusual in studies investigating post-injury care of ankle fractures [21]. Although complications were not an issue, early termination of the study was recommended by the DSMB. An interim analysis was conducted after half of the target enrollment was reached as specified in the study protocol [5]. After interim analysis, the trial was terminated based on the slow inclusion rate and lack of funding. It should be noted that early termination of trials has been shown to overestimate reported benefits, regardless of whether statistical stopping rules are used [2]. To account for this overestimation, an O’Brien post hoc analysis was performed in the present study.

Several features were incorporated in this trial to minimize the amount of bias. Allocation to a treatment group was concealed using a computerized block randomization. In addition, a coordinating researcher performed the follow-up of all patients to keep the loss to follow-up as low as possible to prevent attrition bias. All outcomes were measured in a standardized way using patient-reported questionnaires. The protocol pre-specified our methods and statistical analysis plan, except for the statistical significance bound, for which we decided post hoc to use the conservative O’Brien-Fleming approach [5].

The study was limited by the fact that there was no blinding of patients, treating physicians or outcome assessors; however, it is unclear whether blinding is truly necessary in this pragmatic trial. A potential criticism also mentioned by Dehghan et al. is the potential noncompliance of patients to their designated postoperative care regimen [4, 9]. Patient noncompliance could lead to a higher rate of complications as described previously, although this was absent in the present study [20]. Finally, the rate of randomized to screened patients was low.

## Conclusion

This study suggests that unprotected weight-bearing and mobilization as tolerated results in improved short-term functional outcome and earlier return to work and sports. The postoperative care regimen of unprotected weight-bearing and mobilization as tolerated did not result in an increased rate of complications in adult patients with a supination external rotation type 2–4 ankle fracture without comorbidities. When results are implemented in common clinical practice, careful patient and fracture selection in combination with close observation is warranted.

## Electronic supplementary material

Below is the link to the electronic supplementary material.

Supplementary material 1 (DOCX 28 KB)

## References

[1] Aaronson NK, Muller M, Cohen PD, Essink-Bot ML, Fekkes M, Sanderman R, Sprangers MA, Te VA, Verrips E (1998). Translation, validation, and norming of the Dutch language version of the SF-36 Health Survey in community and chronic disease populations. J Clin Epidemiol.

[2] Bassler D, Briel M, Montori VM, Lane M, Glasziou P, Zhou Q, Heels-Ansdell D, Walter SD, Guyatt GH, Flynn DN, Elamin MB, Murad MH, Abu Elnour NO, Lampropulos JF, Sood A, Mullan RJ, Erwin PJ, Bankhead CR, Perera R, Ruiz CC, You JJ, Mulla SM, Kaur J, Nerenberg KA, Schunemann H (2010). Stopping randomized trials early for benefit and estimation of treatment effects: systematic review and meta-regression analysis. JAMA.

[3] Besselink MG, van Santvoort HC, Buskens E, Boermeester MA, van Timmerman GH, Nieuwenhuijs HM, Bollen VB, van Witteman TLRB, Rosman BJ, Ploeg C, Brink RJ, Schaapherder MA, Dejong AF, Wahab CH, van Laarhoven PJ, van der Harst CJ, van Eijck E, Cuesta CH, Akkermans MA, Gooszen LM (2008). Probiotic prophylaxis in predicted severe acute pancreatitis: a randomised, double-blind, placebo-controlled trial. Lancet.

[4] Braun BJ, Veith NT, Rollmann M, Orth M, Fritz T, Herath SC, Holstein JH, Pohlemann T (2017). Weight-bearing recommendations after operative fracture treatment-fact or fiction? Gait results with and feasibility of a dynamic, continuous pedobarography insole. Int Orthop.

[5] Briet JP, Houwert RM, Smeeing DP, Pawiroredjo JS, Kelder JC, Lansink KW, Leenen LP, van der Zwaal P, van Zutphen SW, Hoogendoorn JM, van Heijl M, Verleisdonk EJ, van Lammeren GW, Segers MJ, Hietbrink F (2015). Weight bearing or non-weight bearing after surgically fixed ankle fractures, the WOW! Study: study protocol for a randomized controlled trial. Trials.

[6] Court-Brown CM, Caesar B (2006). Epidemiology of adult fractures: a review. Injury.

[7] Court-Brown CM, McBirnie J, Wilson G (1998). Adult ankle fractures—an increasing problem?. Acta Orthop Scand.

[8] Daly PJ, Fitzgerald RH, Melton LJ, Ilstrup DM (1987). Epidemiology of ankle fractures in Rochester, Minnesota. Acta Orthop Scand.

[9] Dehghan N, McKee MD, Jenkinson RJ, Schemitsch EH, Stas V, Nauth A, Hall JA, Stephen DJ, Kreder HJ (2016). Early weightbearing and range of motion versus non-weightbearing and immobilization after open reduction and internal fixation of unstable ankle fractures: A Randomized Controlled Trial. J Orthop Trauma.

[10] Finsen V, Saetermo R, Kibsgaard L, Farran K, Engebretsen L, Bolz KD, Benum P (1989). Early postoperative weight-bearing and muscle activity in patients who have a fracture of the ankle. J Bone Joint Surg Am.

[11] Gul A, Batra S, Mehmood S, Gillham N (2007). Immediate unprotected weight-bearing of operatively treated ankle fractures. Acta Orthop Belg.

[12] Heare A, Kramer N, Salib C, Mauffrey C (2017). early versus late weight-bearing protocols for surgically managed posterior wall acetabular fractures. Orthopedics.

[13] Kazemi N, Archdeacon MT (2012). Immediate full weightbearing after percutaneous fixation of anterior column acetabulum fractures. J Orthop Trauma.

[14] Keene DJ, Williamson E, Bruce J, Willett K, Lamb SE (2014). Early ankle movement versus immobilization in the postoperative management of ankle fracture in adults: a systematic review and meta-analysis. J Orthop Sports Phys Ther.

[15] Kubiak EN, Beebe MJ, North K, Hitchcock R, Potter MQ (2013). Early weight bearing after lower extremity fractures in adults. J Am Acad Orthop Surg.

[16] Lauge-Hansen N. Ankelbrud I. Genetisk diagnose og reposition. Copenhagen MD thesis. Munksgaard 1942.

[17] Lehtonen H, Jarvinen TL, Honkonen S, Nyman M, Vihtonen K, Jarvinen M (2003). Use of a cast compared with a functional ankle brace after operative treatment of an ankle fracture. A prospective, randomized study. J Bone Joint Surg Am.

[18] Lin CW, Donkers NA, Refshauge KM, Beckenkamp PR, Khera K, Moseley AM (2012). Rehabilitation for ankle fractures in adults. Cochrane Datab Syst Rev.

[19] Michelson JD (1995). Fractures about the ankle. J Bone Joint Surg Am.

[20] Miller AG, Margules A, Raikin SM (2012). Risk factors for wound complications after ankle fracture surgery. J Bone Joint Surg Am.

[21] Moseley AM, Beckenkamp PR, Haas M, Herbert RD, Lin CW (2015). Rehabilitation after immobilization for ankle fracture: the EXACT Randomized Clinical Trial. JAMA.

[22] Nash CE, Mickan SM, Del Mar CB, Glasziou PP (2004). Resting injured limbs delays recovery: a systematic review. J Fam Pract.

[23] Nortunen S, Leskela HV, Haapasalo H, Flinkkila T, Ohtonen P, Pakarinen H (2017). Dynamic stress testing is unnecessary for unimalleolar supination-external rotation ankle fractures with minimal fracture displacement on lateral radiographs. J Bone Joint Surg Am.

[24] Olerud C, Molander H (1984). A scoring scale for symptom evaluation after ankle fracture. Arch Orthop Trauma Surg.

[25] Schepers T, De Vries MR, Van Lieshout EM, Van der Elst M (2013). The timing of ankle fracture surgery and the effect on infectious complications; a case series and systematic review of the literature. Int Orthop.

[26] Simanski CJ, Maegele MG, Lefering R, Lehnen DM, Kawel N, Riess P, Yucel N, Tiling T, Bouillon B (2006). Functional treatment and early weightbearing after an ankle fracture: a prospective study. J Orthop Trauma.

[27] Smeeing DPJ, Briet JP, van Kessel CS, Segers MJM, Verleisdonk EJMM, Leenen LPH, Houwert RM, Hietbrink F (2017). Factors associated with wound- and implant related complications following surgical treatment of ankle fractures. J Foot Ankle Surg.

[28] Smeeing DP, Houwert RM, Briet JP, Kelder JC, Segers MJ, Verleisdonk EJ, Leenen LP, Hietbrink F (2015). Weight-bearing and mobilization in the postoperative care of ankle fractures: a systematic review and meta-analysis of randomized controlled trials and cohort studies. PLoS One.

[29] Stadhouder A, Buskens E, de Klerk LW, Verhaar JA, Dhert WA, Verbout AJ, Vaccaro AR, Oner FC (2008). Traumatic thoracic and lumbar spinal fractures: operative or nonoperative treatment: comparison of two treatment strategies by means of surgeon equipoise. Spine (Phila Pa 1976).

[30] Stadhouder A, Oner FC, Wilson KW, Vaccaro AR, Williamson OD, Verbout AJ, Verhaar JA, de Klerk LW, Buskens E (2008). Surgeon equipoise as an inclusion criterion for the evaluation of nonoperative versus operative treatment of thoracolumbar spinal injuries. Spine J.

[31] Swart E, Bezhani H, Greisberg J, Vosseller JT (2015). How long should patients be kept non-weight bearing after ankle fracture fixation? A survey of OTA and AOFAS members. Injury.

[32] Tan EW, Sirisreetreerux N, Paez AG, Parks BG, Schon LC, Hasenboehler EA (2016). Early weightbearing after operatively treated ankle fractures: a biomechanical analysis. Foot Ankle Int.

[33] van Laarhoven CJ, Meeuwis JD, van dW (1996). Postoperative treatment of internally fixed ankle fractures: a prospective randomised study. J Bone Joint Surg Br.

[34] van Santvoort HC, Besselink MG, Bakker OJ, Hofker HS, Boermeester MA, Dejong CH, van Goor H, Schaapherder HC, van Eijck CH, Bollen TL, van Ramshorst B, Nieuwenhuijs VB, Timmer R, Lameris JS, Kruyt PM, Manusama ER, van der Harst E, van der Schelling GP, Karsten T, Hesselink EJ, van Laarhoven CJ, Rosman C, Bosscha K, de Wit RJ, Houdijk AP (2010). A step-up approach or open necrosectomy for necrotizing pancreatitis. N Engl J Med.

[35] Vioreanu M, Dudeney S, Hurson B, Kelly E, O’Rourke K, Quinlan W (2007). Early mobilization in a removable cast compared with immobilization in a cast after operative treatment of ankle fractures: a prospective randomized study. Foot Ankle Int.

[36] World Medical Association. WMA Declaration of Helsinki: ethical principles for medical research involving human subjects. 2016. https://www.wma.net/policies-post/wma-declaration-of-helsinkiethical-principles-for-medical-research-involving-human-subjects/. Accessed 4 Jan 2016

