# Supplementary material for: Weight-bearing or non-weight-bearing after surgical treatment of ankle fractures: a multicenter randomized controlled trial
Source: Eur J Trauma Emerg Surg. 2018 Sep 24;46(1):121–30. doi: 10.1007/s00068-018-1016-6 (PMC7026225; doi:10.1007/s00068-018-1016-6)
Supplement: Supplementary file 1 — Supplementary material 1 (DOCX 28 KB) [file 68_2018_1016_MOESM1_ESM.docx]

**Appendix 1: In- and exclusion criteria and reasons for not participating**

|  | AZN | DHU | HMC | EZH | **total** |
| --- | --- | --- | --- | --- | --- |
| **Exclusion criteria** |  |  |  |  |  |
| ● pre-existent impaired mobility | 1 | 3 | 0 | 1 | **5** |
| ● pre-existent impaired cognitive disability | 4 | 2 | 9 | 5 | **20** |
| ● expected insufficient stable fracture fixation with standard surgical technique | 0 | 0 | 0 | 0 | **0** |
| ● necessity of a syndesmosis screw | 13 | 3 | 16 | 7 | **39** |
| ● posterior malleolar fragment requiring operative fixation | 15 | 2 | 26 | 12 | **55** |
| ● body mass index >30 | 18 | 6 | 4 | 5 | **33** |
| ● diabetes mellitus | 7 | 8 | 10 | 3 | **28** |
| ● polytrauma patients | 0 | 0 | 1 | 2 | **3** |
| ● Gustilo 2 and 3 open fractures | 4 | 3 | 5 | 1 | **14** |
| ● inability to comply with one of the treatment groups | 4 | 3 | 4 | 3 | **14** |
| ● inability to comply with follow-up | 2 | 3 | 3 | 2 | **10** |
| ● inability to comply with follow-up because of language | 2 | 1 | 5 | 2 | **10** |
| ● too severe soft tissue damage and/or initial use of external fixation | 0 | 3 | 6 | 5 | **14** |
| ● pregnancy | 0 | 1 | 0 | 0 | **1** |
| ● other injuries dictating postoperative care | 3 | 3 | 1 | 6 | **13** |
| ● insufficient stable fracture fixation established during surgery as judged by the treating surgeon | 1 | 1 | 3 | 0 | **5** |
|  |  |  |  |  |  |
| **No participation** |  |  |  |  |  |
| ● trial not discussed | 36 | 9 | 92 | 79 | **216** |
| ● refusal to participate | 6 | 13 | 13 | 4 | **36** |
|  |  |  |  |  |  |
| **Included** | 31 | 60 | 18 | 6 | **115** |

AZN = St Antonius

DHU = Diakonessenhuis

HMC = Haaglanden Medisch Centrum

EZH = Elisabeth-TweeSteden

**Appendix 2: Follow-up rate per time-point**

| Hospital | 2 weeks | | 6 weeks | | 12 weeks | | 6 months | | 1 year | |
| --- | --- | --- | --- | --- | --- | --- | --- | --- | --- | --- |
| AZN | 31/31 | 100% | 28/31 | 90.3% | 29/31 | 93.5% | 26/31 | 83.9% | 29/31 | 93.5% |
| DHU | 60/60 | 100% | 54/60 | 90.0% | 57/60 | 95.0% | 43/60 | 71.7% | 55/60 | 91.7% |
| HMC | 18/18 | 100% | 12/18 | 66.7% | 12/18 | 66.7% | 11/18 | 61.1% | 16/18 | 88.9% |
| EZH | 6/6 | 100% | 4/6 | 66.7% | 3/6 | 50.0% | 1/6 | 16.7% | 6/6 | 100% |
| **Total** | **115/115** | **100%** | **98/115** | **85.2%** | **101/115** | **87.8%** | **81/115** | **70.4%** | **106/115** | **92.2%** |

AZN = St Antonius

DHU = Diakonessenhuis

HMC = Haaglanden Medisch Centrum

EZH = Elisabeth-TweeSteden

**Appendix 3: Group comparisons of Olerud Molander Ankle Score, time to return to work and sports**

| **Unprotected weight-bearing (n=42) vs Protected weight-bearing (n=36)** | | | |
| --- | --- | --- | --- |
|  | Mean difference | 95% CI of the difference | p-value |
| Olerud Molander score 6 weeks | 9.4 | (-.39 to 19.2) | 0.059 |
| Olerud Molander score 12 weeks | 3.6 | (-4.7 to 11.8) | 0.391 |
| Olerud Molander score 6 months | -0.4 | (-9.5 to 8.7) | 0.925 |
| Olerud Molander score 1 year | -2.2 | (-9.6 to 5.1) | 0.542 |
| Time to return to work in weeks˟ | -1.6 | (-3.6 to 0.3) | 0.105 |
| Time to return to sports in weeks˟ | -3.8 | (-7.6 to 0) | 0.048 |
| **Unprotected weight-bearing (n=42) vs Unprotected non-weight-bearing (n=37)** | | | |
|  | Mean difference | 95% CI of the difference | p-value |
| Olerud Molander score 6 weeks | 15.4 | (5.3 to 25.6) | 0.004 |
| Olerud Molander score 12 weeks | 4.3 | (-5.0 to 13.6) | 0.357 |
| Olerud Molander score 6 months | 4.6 | (-5.5 to 14.7) | 0.362 |
| Olerud Molander score 1 year | -1.9 | (-8.3 to 4.6) | 0.564 |
| Time to return to work in weeks˟ | -2.9 | (-5.1 to -0.7) | 0.010 |
| Time to return to sports in weeks˟ | -5.2 | (-8.0 to -2.4) | <0.001 |
| **Protected weight-bearing (n=36) vs Unprotected non-weight-bearing (n=42)** | | | |
|  | Mean difference | 95% CI of the difference | p-value |
| Olerud Molander score 6 weeks | 6.0 | -4.7 to 16.7 | 0.265 |
| Olerud Molander score 12 weeks | 0.7 | -8.0 to 9.5 | 0.866 |
| Olerud Molander score 6 months | 5.0 | -3.8 to 13.9 | 0.259 |
| Olerud Molander score 1 year | 0.4 | -6.1 to 6.9 | 0.908 |
| Time to return to work in weeks˟ | -1.3 | -3.8 to 1.2 | 0.312 |
| Time to return to sports in weeks˟ | -1.4 | -5.5 to 2.7 | 0.492 |

Independent Samples T-test was used. Equal variances were not assumed.

**Appendix 4: Anecdotes of unprotected weight-bearing participants**

- Two weeks postoperatively, a patient reported that he had been catching Pokemons and thereby walking about 2 kilometres a day and riding his bike for 10 kilometres a day.
- Two weeks postoperatively, a patient arrived by motorcycle for a follow-up visit.
- Six weeks postoperatively, a patient could already walk 10 consecutive kilometres.
- Four and a half weeks postoperatively, a patient was working and playing sports again.
